# High rates of growth recorded for hawksbill sea turtles in Anegada, British Virgin Islands

**DOI:** 10.1002/ece3.1018

**Published:** 2014-03-13

**Authors:** Lucy A Hawkes, Andrew McGowan, Annette C Broderick, Shannon Gore, Damon Wheatley, Jim White, Matthew J Witt, Brendan J Godley

**Affiliations:** 1 Centre for Ecology & Conservation, College of Life and Environmental Sciences, University of Exeter, Penryn CampusPenryn, TR10 9EZ, U.K; 2 Conservation and Fisheries Department, Ministry of Natural Resources and LabourPO Box 3323, Road Town, Tortola, British Virgin Islands; 3 The SettlementAnegada, British Virgin Islands; 4 Environment and Sustainability Institute, University of Exeter, Penryn CampusPenryn, Cornwall, TR10 9EZ, U.K

**Keywords:** Caribbean, conservation, growth rates, hawksbill turtle, sex ratio, sexual maturity

## Abstract

Management of species of conservation concern requires knowledge of demographic parameters, such as rates of recruitment, survival, and growth. In the Caribbean, hawksbill turtles (*Eretmochelys imbricata*) have been historically exploited in huge numbers to satisfy trade in their shells and meat. In the present study, we estimated growth rate of juvenile hawksbill turtles around Anegada, British Virgin Islands, using capture–mark–recapture of 59 turtles over periods of up to 649 days. Turtles were recaptured up to six times, having moved up to 5.9 km from the release location. Across all sizes, turtles grew at an average rate of 9.3 cm year^−1^ (range 2.3–20.3 cm year^−1^), and gained mass at an average of 3.9 kg year^−1^ (range 850 g–16.1 kg year^−1^). Carapace length was a significant predictor of growth rate and mass gain, but there was no relationship between either variable and sea surface temperature. These are among the fastest rates of growth reported for this species, with seven turtles growing at a rate that would increase their body size by more than half per year (51–69% increase in body length). This study also demonstrates the importance of shallow water reef systems for the developmental habitat for juvenile hawksbill turtles. Although growth rates for posthatching turtles in the pelagic, and turtles larger than 61 cm, are not known for this population, the implications of this study are that Caribbean hawksbill turtles in some areas may reach body sizes suggesting sexual maturity in less time than previously considered.

## Introduction

The effective recovery of exploited populations depends on a variety of demographic factors, including survival and growth to maturity of existing individuals and recruitment of new individuals to the population (Lotze et al. 2011; Mills 2013a). Key in managing species recovery, therefore, is an understanding of these factors and the dynamic interactions between them. Unfortunately, for many populations of species of conservation concern, such data may be lacking, leading to a substitution of data collected from other, better-studied species or populations, which may be inappropriate (Caro et al. 2005; Schtickzelle et al. 2005; Githirua et al. 2007; Peck et al. 2008). Recent work, however, has suggested that demographic parameters can vary among populations of the same species within geographic regions and thus may not follow expected patterns from life-history theory (Johnson et al. 2010). Thus, there is potential for models of population recovery trajectories to misrepresent reality where data are sparse. This may be compounded by modern anthropogenic influences such as climate change (Coulson et al. 2001; Nilsen et al. 2009).

Knowledge of growth rates can inform effective conservation practice (Mills 2013c). For example, assessing whether the beneficial effects of conservation strategies are realized in the population at large may depend on the period to sexual maturity, and thus on growth rate. For the seven species of marine turtles, which are of conservation concern, conservation interventions generally take place at the nesting beach (e.g., protection of incubating eggs and nesting females) or at sea (e.g., alterations to fishing gears to reduce bycatch and exclusion of fisheries from marine protected areas). Conservation at sea should have rapid benefits to the population (reducing mortality in reproductively active individuals), but the benefits of conservation on the nesting beach will be realized only after the hatchling cohort protected in a given year reach sexual maturity (Crouse et al. 1987). In addition, demographic modeling can help estimate the impact of harvest of endangered species, contributing to stock assessment to inform setting of catch quotas (Mills 2013b). Several nations in the Caribbean still host small-scale, legal, artisanal level, fisheries for hawksbill turtles (Richardson et al. 2006), so data are needed to inform current harvest, as well as any future harvest that were re-instated.

In the Caribbean, hawksbill sea turtles (*Eretmochelys imbricata*) were once abundant and supplied a global trade for “tortoiseshell” (the attractive scute plates that make up the hawksbill turtles carapace (Meylan 1999; McClenachan et al. 2006)). Overharvest led to reduction in the Caribbean population, which is thought to remain at relictual levels today (McClenachan et al. 2006). Fortunately, increases in nesting numbers have been observed for several nesting rookeries of the species (Beggs et al. 2007; Kamel and Delcroix 2009; Allen et al. 2010), and satellite tracking is now yielding much information about the spatio-temporal distribution of adult hawksbill turtles in the Caribbean (e.g., (Meylan et al. 2011; Hawkes et al. 2012; Moncada et al. 2012)). Despite this, there is still a paucity in the understanding of the demography of hawksbill turtles – including growth rates and age at sexual maturity (Meylan et al. 2011).

## Aims

In this study, we set out to describe rates of growth, in both body size and mass, for juvenile hawksbill turtles around Anegada, British Virgin Islands, an important Caribbean foraging habitat (McGowan et al. 2008).

## Materials and Methods

### Turtle capture

**Figure 1:**
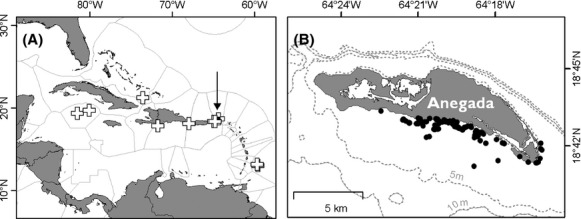
Map showing (A) the location of the British Virgin Islands in the Caribbean (black arrow) and the locations of other studies of growth rates in Caribbean hawksbill turtles (white crosses). (B) the locations of 134 captures of juvenile hawksbill turtles around Anegada, British Virgin Islands (black dots), gray dashed lines show bathymetric contours of 5, 10 (labeled) and 15 m (not labeled).

Hawksbill turtles were captured as part of an in-water sampling program using the rodeo technique (Limpus 1981) in waters around Anegada, British Virgin Islands (Fig. 1; (McGowan et al. 2008; Hawkes et al. 2013)). Surveys took place over 109 irregularly spaced days between the 16 November 2003 and the 8 August 2006, comprising a total effort of 543 h. Surveying for turtles took place in waters shallow enough for capture to take place (generally <20 m depth) and within the vicinity of reefs (see also (McGowan et al. 2008; Witt et al. 2010)). Turtles were hand captured using the Rodeo technique when they were either sighted surfacing to breathe or resting on the sea floor. On capture, turtles were flipper tagged (using Inconel tags), PIT (passive integrated transponder) tagged, and biometric measurements taken, including carapace length (from anterior notch to posterior tip, to the nearest 0.1 cm), carapace width (curved measurements for both length and width using a tape measure and straight measurements with vernier callipers; Bolten 1999, also to the nearest 0.1 cm), and body mass (using spring balances accurate to the nearest 0.3%). All measurements were averages of three measurements made by either MW or AM to reduce interindividual variation. GPS (Global Positioning System) location was recorded for 74 captures. On occasions where curved carapace length (CCL) measurements were not collected (*n* = 26 turtles), they were estimated using straight carapace length (CCL = 1.1 × SCL + 0.1; *R*^2^ = 0.99, *t* = 171.0, *P* < 0.01 in 267 turtles for which we had both measurements), accepting that inaccuracies in the straight carapace length measurements would be carried forward to CCL estimates. Blood samples were collected from turtles for analysis of testosterone and oestradiol-17*β* (results reported in (Hawkes et al. 2013)), and hormone values compared with thresholds reported in Geis et al. (2003), Diez and van Dam (2003) and Blanvillain et al. (2008) to estimate sex of captured individuals.

### Growth estimation

Growth rate (in centimeters per year; cm year^−1^) was estimated as the increment in CCL between captures divided by the days elapsed since initial capture, multiplied by 365 days of a year. For turtles recaptured after short intervals, measurement errors may be proportionally larger and may lead to spurious estimations of growth. We accounted for this using only growth rate estimations from recapture intervals of 60 days or more because this should have yielded growth increments of at least approximately 0.5 cm (based on previously published values (Witzell 1980; Bjorndal and Bolten 1988; Limpus 1992; Boulon 1994; Chaloupka and Limpus 1997; Leon and Diez 1999; Diez and van Dam 2002; Shima et al. 2004; Beggs et al. 2007; Blumenthal et al. 2009a; Bjorndal and Bolten 2010; Krueger et al. 2011; Bell and Pike 2012; Snover et al. 2013; Wood et al. 2013)), and which should have been well within the range of measurement accuracy reported in other studies (Bjorndal and Bolten 1988). We assumed that recapture and resampling of turtles did not affect subsequent growth (Bjorndal et al. 2010). We also calculated mass gain (in kilogrammes per year, kg year^−1^) for the same turtles recaptured after more than 60 days, calculated as the mass change in kilograms divided by the days since initial capture, multiplied by 365. In this study, growth rate refers to gain in carapace length, and mass gain refers to change in body mass.

### Distance between captures

The distance between individual turtle captures was calculated as the hypotenuse of the triangle described by the paired northings and eastings, for each release and recapture, respectively.

### Environmental parameters

Sea surface temperature data were extracted from the MODIS Aqua satellite (http://modis.gsfc.nasa.gov) 8-day mean data product for each day on which hawksbill turtles were recaptured in the present study, unless cloud cover obscured the site (which happened on seven capture days). Data were also extracted from MODIS for each of the study sites for which growth rates have been previously reported for hawksbill turtles (Table 1) and averaged over the duration of the study period reported.

**Table 1:** Reported values for annual growth rates (in cm year^−1^) of adult and juvenile hawksbill turtles grouped by size classes (10 cm carapace length increments) from the Caribbean Sea and Pacific Ocean, sample size (*n*) shown in brackets.

Life stage	Location	20–30 cm	30–40 cm	40–50 cm	50–60 cm	60–70 cm	>70 cm	Method	Interval (days)	Measurement type (cm)	References
Atlantic & Caribbean											
Adult & Juvenile	USA	–	–	4.49 (3)	3.42 (8)	2.34 (10)	1.68 (3)	Mean	365	SCLn-t	Wood et al. (2013)
Adult & Juvenile	Bahamas	–	15.60 (1)	7.35 (15)	5.21 (16)	3.14 (5)	–	Mean	337	SCLn-t	Bjorndal and Bolten (2010)^1^
Adult & Juvenile	Bahamas	–	15.70 (1)	5.90 (1)	–	2.60 (3)	–	Mean	0	SCLn-n	Bjorndal and Bolten (1988)^1^
Adult & Juvenile	Barbados	–	3.28	1.84	0.88	0.43	0.13	(–)	330	CCLn-t	Krueger et al. (2011)^2^
Adult	Barbados	–	–	–	–	–	0.40 (1274)	Mean	365	CCLn-t	Beggs et al. (2007)
Juvenile	Cayman Is.	3.46 (3)	3.09 (21)	2.67 (10)	1.93 (3)	–	–	Mean	11	SCLn-t	Blumenthal et al. (2009a)
Juvenile	Dominican Rep.	5.26 (12)	6.47 (10)	–	–	–	–	Initial	45	SCLn-t	Leon and Diez (1999)
Juvenile	Puerto Rico	4.22 (99)	4.49 (45)	3.86 (29)	3.02 (10)	2.03 (5)	0.97 (6)	Mean	292	SCLn-t	Diez and van Dam (2002)^3^
Juvenile	USVI	–	4.80 (3)	3.30 (5)	2.80 (3)	2.60 (1)	–	Initial	93	SCLn-t	Boulon (1994)
Juvenile	BVI (Anegada)	10.84 (20)	8.91 (45)	8.69 (16)	6.14 (4)	–	–	Initial	60	CCLn-t	Present study
Pacific											
Adult & Juvenile	Australia (GBR)	–	–	–	–	1.67	1.25	Mean	337	CCLn-t	Bell and Pike (2012)^2^
Juvenile	Australia (GBR)	–	–	1.29	1.90	1.83	0.96	Mean	337	CCLn-t	Chaloupka and Limpus (1997)^2^^,^^4^
	Australia	–	–	1.39	2.22	1.92	1.01	Mean	337	CCLn-t	Limpus (1992)^1^
Adult & Juvenile	Japan	–	–	2.20	–	–	–	(–)	(–)	SCL*	Shima et al. (2004)^2^
Adult & Juvenile	Hawaiian Is.	3.80 (21)	4.40 (22)	5.16 (17)	2.24 (11)	2.24 (14)	3.60 (18)	Mean	N/A	SCLn-t	Snover et al. (2013)
Juvenile	Western Samoa	1.29 (6)	1.88 (3)	0.72 (8)	–	–	–	Mean	N/A	SCLn-t	Witzell (1980)^5^

Methodology used to relate growth rates to turtle size indicated as mean (mean of capture and recapture measurements) or initial (initial capture measurement only related to growth rate). Some studies did not detail which methodology was used. Minimum interval used to estimate growth rate (in days) and carapace measurement type (SCL, Straight Carapace Length; CCL, Curved Carapace Length, n-n, anterior notch to posterior notch; n-t, anterior notch to posterior tip) is also indicated.

1 Bjorndal and Bolten (2010) report a total of five hawksbill turtles, which are included in Bjorndal and Bolten (1988).

2 It was not possible to extract sample sizes for this study.

3 Grouped data for three sites.

4 Grouped data for males and females.

5 Turtles reared in captivity.

6 This study did not state whether SCL measurements were n-t or n-n.

### Age at sexual maturity

We estimated the age at sexual maturity for hawksbill turtles in the British Virgin Islands by fitting an exponential decay function to a plot of carapace length at first capture against growth rate using a least squares approach. We iteratively derived the optimal values for the input parameters initial quantity (*N*_0_) and lambda (*λ*) until the difference between observed and predicted values was minimized. We then extracted the time taken in days for a turtle to grow 1 cm in carapace length for 1 cm increments between the range of carapace sizes measured in the present study and summed them to obtain the total time that would have been needed for an average turtle to grow from our minimum to our maximum measured carapace length. We then extrapolated outside the data range to obtain a coarse first estimate of the time taken for a hatchling hawksbill turtle to reach sexual maturity (defined as 67 cm CCL (Meylan et al. 2011)). We then repeated this process for mass gain.

### Comparison with other foraging aggregations

Because growth rates are unlikely to remain constant across a range of body sizes (i.e., larger individuals might be expected to grow more slowly than smaller individuals as they reach sexual maturity), we compared growth rates in this study with previously published growth rates for 10-cm-wide curved carapace length bins, rather than using overall mean values across all sizes encountered by each study. These were extracted from published papers (Bjorndal and Bolten 1988, 2010; Boulon 1994; Leon and Diez 1999; Diez and van Dam 2002; Beggs et al. 2007; Blumenthal et al. 2009a; Krueger et al. 2011) by digitizing published plots, extracting raw values, and grouping growth rates into 10-cm-wide bins. Unfortunately, the published studies did not all use the same methodology to relate growth to carapace size, with some studies relating to the size at initial release and others relating to the mean size between release and recapture. It was thus not possible to standardize reported growth rates to growth bins, and we therefore detail the methodologies used in each study in Table 1.

### Statistics

Prior to testing, all data (growth rates, mass gain, carapace size, sea surface temperature, and sex) were tested for normality using Shapiro–Wilks test. Correlation tests were carried out using Pearson product moment correlation coefficient, and general linear mixed modeling used to test relationships among factors, controlling for turtle ID as a random effect (using the package ‘nlme’). All graphs were produced and analyses undertaken in R.

## Results

### Turtle capture

**Figure 2:**
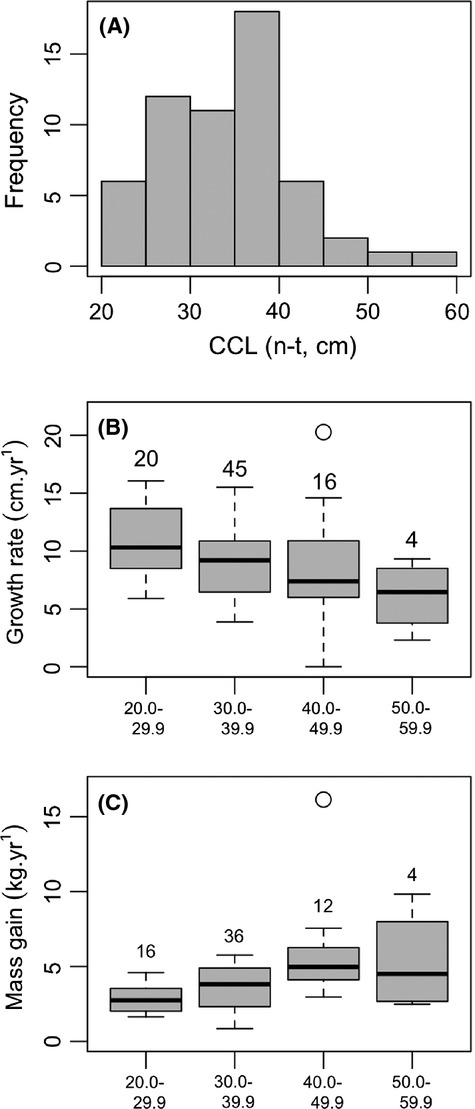
Plots showing (A) frequency histogram of carapace length (CCL, n-t) for all hawksbill turtles on initial capture, boxplots of (B) growth rate and (C) mass gain of juvenile hawksbill turtles in Anegada, showing rates for four size classes of turtles at first capture (in centimeters), n individuals noted above each boxplot. Gray box shows interquartile range, solid black line shows median value. Circles show statistical outlier value. Growth rates decreases significantly with body size, while mass gain increases.

**Figure 3:**
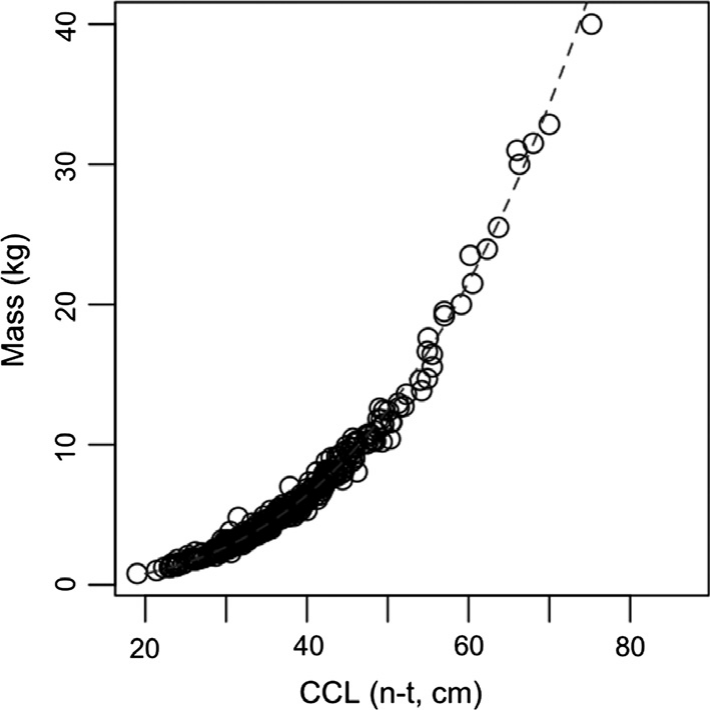
Plot showing mass of juvenile hawksbill turtles captured around Anegada, British Virgin Islands, against carapace length (CCL) measured from the nuchal notch to the posterior tip of the carapace (n-t) at first capture. Dashed line shows cubic smoothing spline (mass = 0.0001 × body length^3^).

During the study period, we made 389 captures of hawksbill turtles on the south coast of Anegada, comprising a total of 249 individuals, which were measured and released with flipper and PIT tags immediately after capture (Fig. 1). Most turtles (*n* = 176) were not subsequently seen again, but 73 were recaptured between one (*n* = 41) and six (*n* = 1) more times. Turtles ranged from minimum 22.3 cm CCL at initial capture to 60.5 cm CCL at recapture (Fig. 2A) and from 1.22 kg at initial capture to 21.5 kg at recapture and were thus all considered juvenile. Turtle mass and CCL were significantly and exponentially correlated (Pearson's correlation test on log-transformed data *ρ* = 0.99, *P* < 0.01; Fig. 3). After removing recaptures that occurred 60 days or less after initial capture, the remaining 85 estimations of growth rate (from 59 turtles) were recorded on average 239 days apart (mean value ± 147 days SD, range 63–649 days).

### Growth

Juvenile hawksbill turtles grew at 9.3 cm year^−1^ (mean ± 3.2 SD, range 2.3–20.3; Fig. 2B). The greatest rate of growth (20.3 cm year^−1^) was recorded for one turtle of 40 cm CCL at initial capture, which was recaptured after 108 days at liberty having gained 6 cm carapace length. After release, it was subsequently recaptured 38 days later having sustained the same rate of growth over that period too. The greatest recorded change in carapace size between captures was observed for a hawksbill of 36 cm CCL, which was recaptured after 611 days at liberty having gained 18.2 cm CCL (equivalent to a growth rate of 10.9 cm year^−1^). Seven turtles grew at a rate that would have increased CCL by more than half annually, and one turtle grew at a rate that would have increased carapace length by 70% per year. These phenomenal growth rates are, to our knowledge, among the fastest recorded in wild hawksbill turtles.

Juvenile hawksbill turtles gained mass at an average of 3.9 kg year^−1^ (±2.2 SD, range 0.9–16.1; Fig. 2C). The greatest rate of mass gain (16.1 kg year^−1^) was recorded for one turtle of 11.8 kg at initial capture. It was recaptured 130 days later weighing 17.6 kg (and was also one of the five fastest growing turtles, by carapace length). The greatest change in mass between captures was for a turtle that weighed 4.5 kg at initial capture and was recaptured after 611 days having gained 9.4 kg (equivalent to a growth rate of 5.6 kg year^−1^; the same turtle detailed above with the greatest change in carapace size). It was not possible to test for differences in growth rate or mass gain between male and female turtles as there were insufficient males (four of 53 turtles for which sex was determined).

**Figure 4:**
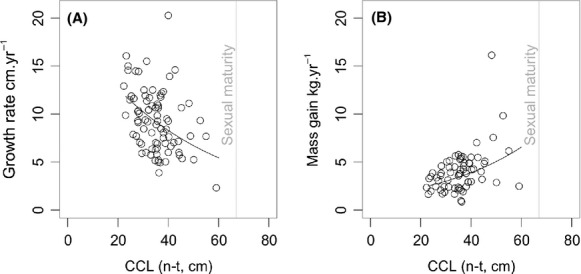
Plots showing fitted model (black line) and raw data (open circles) of (A) growth rate and (B) mass gain as a function of carapace length at first capture. Dashed gray vertical line shows approximate size at sexual maturity for hawksbill turtles (67 cm; (Meylan et al. 2011)).

Growth rate differed significantly with initial carapace size (GLMM: *Χ*_1_ = 8.2, *P* < 0.01), such that smaller turtles grew faster than larger turtles (Figs. 2B, 4A). For example, smaller turtles between 20.0 and 29.9 cm carapace length grew on average at 10.8 cm year^−1^, while larger turtles between 50.0 and 59.9 cm carapace length grew at approximately half this rate (median 6.1 cm year^−1^). However, mass gain showed the opposite pattern, such that larger turtles put on mass more rapidly than did smaller turtles (GLMM: *Χ*_1_ = 18.7, *P* < 0.0001; Figs. 2C, 4B). For example, turtles between 20.0 and 29.9 cm carapace length put on mass at an average of 2.8 kg year^−1^, whereas turtles between 50.0 and 59.9 cm carapace length put on mass almost twice as fast at 5.3 kg year^−1^.

### Distance between captures

Turtles were recaptured on average only 0.5 km away from their release site (±1.1 km SD, *n* = 45 recapture distances), with one turtle recaptured only 14 m away from its original capture location where it was released 91 days earlier. One turtle moved as far as 5.9 km away from the original capture location, being recaptured 360 days later.

### Environmental parameters

Turtle growth in the present study did not appear to be affected by sea surface temperature (mean, minimum, or maximum temperature over the recapture interval, GLMM: *Χ*_1_ = 0.007, *P* = 0.94). There was also no correlation between growth rate for other studies reported in the literature and mean, minimum, maximum, or variation in sea surface temperatures extracted for each study site (Pearson's correlation *P* > 0.05). Sea surface temperatures varied by only 4.5°C around Anegada over the entire study period (range 25.3–29.8°C), although the actual temperatures experienced by turtles, which would have differed from the surface when turtles were diving, is not known.

### Age at sexual maturity

If the model presented in this study was realistic (and further work is required before this can be ascertained), the results of the present study would suggest that hawksbill turtles in the British Virgin Islands could grow from 22 cm CCL to 60 cm CCL in as little as 4 years and 11 months (1804 days; exponential decay model with parameters *N*_0_ = 19.3 and *λ *= −0.02; Fig. 4A, B). It would also suggest that turtles could grow from hatchling size to 67 cm carapace length (Meylan et al. 2011) in 7 years and 8 months.

### Comparison with other foraging aggregations

With the exception of two individuals reported in Bjorndal and Bolten (1988, 2010), hawksbill turtles in this study grew faster in all size classes than any previously reported turtles. It was only possible to directly compare the present results with five studies that used the same measurement type (CCLn-t) to measure growth rates (Limpus 1992; Chaloupka and Limpus 1997; Beggs et al. 2007; Krueger et al. 2011; Bell and Pike 2012). The present growth rates were between 170% (size class 30–40 cm) and 390% (size class 40–50 cm) greater than these, with turtles growing up to 5.6, 7.2, and 4.5 cm year^−1^ faster in each of the size classes 20–30 cm, 30–40 cm, and 40–50 cm, respectively. In the other ten studies in which growth rate was expressed as straight carapace length, equations from Limpus 1992 and van Dam and Diez 1998 (Limpus 1992; van Dam and Diez 1998) were used to convert SCL measurements into CCL. CCL estimates from these two equations differed by up to 9 cm for an 80 cm CCL hawksbill turtle, so should be used with caution. Nevertheless, regardless of which equation was used, growth rate was faster in the present study for all size classes except for turtles between 30 and 40 cm CCL, which was due to two individuals reported in Bjorndal and Bolten (1988, 2010), and the rest of the turtles in their study in that size class grew more slowly than the present study.

## Discussion

Growth rates in the present study are among the greatest for hawksbill turtles reported so far (Table 1) and are even higher than rates reported for hawksbill turtles in captivity, which are unlikely to be resource limited (Witzell 1980). Eleven turtles grew faster than captive green sea turtles fed on a high-protein diet in the Cayman Island turtle farm (*Chelonia mydas*: up to 12 cm year^−1^, (Bjorndal et al. 2013)). Even average growth rates (9.30 cm year^−1^) are higher than previously reported values, and half the turtles (*n* = 31 of 59 turtles total) grew at rates contingent with increasing carapace length by approximately 25% annually. Rates of mass gain, which have rarely been reported for hawksbill turtles, were contingent with turtles almost doubling in body mass annually, but were not greater than for captive green turtles (Bjorndal et al. 2013). The relationship between carapace length and mass gain may be more complex than that between carapace length and growth rate as our study included some individuals with unusually high rates of mass gain (Fig. 4B). While it seems unlikely, this could be due to measurement error (the measurements deviate from the fitted model by approximately 10 kg) and it is worthy of future research attention. For example, it is possible that such differences in rates of mass gain could be related to stochasticity in the size at which individuals recruit to coastal waters from the oceanic developmental phase, such that individuals that recruit at larger sizes (which may in some cases be later life stages) may exhibit compensatory growth.

While measurement error could lead to erroneous estimates of rates of growth over short intervals, the greatest rate of growth and mass gain were recorded over a 611 day interval and are therefore unlikely to be highly inaccurate. Further, measurements of carapace length and body mass were extremely closely correlated, following the square-cube law. It is unclear why our growth rates are generally greater than previously reported values for this species, but the methodology used and interpretation of results do not differ significantly to previous studies such that could explain the difference. Measurement of carapace size is a standard technique in sea turtle research, and we do not expect that there can be improvements made to such measures that might have influenced the difference between our results and previously published work. Nevertheless, it appears that growth rates in the British Virgin Islands are particularly high.

It may not be surprising that the present study did not highlight a relationship between growth rate and ambient temperature for several reasons. First, we used sea surface temperature data products, whereas turtles likely experienced a range of different subsurface temperatures as they exploit different depths while foraging and resting (Witt et al. 2010). Data describing ocean temperature at depth are not available over wide spatial scales. Second, single averaged estimates of sea surface temperature were extracted for the period between release and recapture and correlated against growth rate – integrating at least 63 days and up to 649 days of surface temperature variability. For previously published literature, estimates of sea surface temperature were extracted for the entire study period, which sometimes spanned several years. Clearly, this process would have smoothed all but the most dramatic variations in growth rates among individuals and studies. The relationship between ambient temperature and growth rate in hawksbill turtles may therefore be best studied in captive, controlled conditions and is outside of the feasibility of the present study.

The present study suggests that turtles can grow over half their adult body size (38 cm, from 22 to 60 cm CCL, where mean adult size = 70 cm CCL (Witzell 1980; Meylan et al. 2011)) in as little as 4 years and 11 months. In order to estimate age at sexual maturity, however, data are needed for growth rates of turtles both smaller than 22 cm CCL and larger than 60 cm CCL (outside the range measured in the present study). Unfortunately, very little is known of neonate or posthatchling stage marine turtles (the ‘lost years’; (Carr et al. 1978)), but as these small juveniles are thought to occupy the pelagic zone, where foraging opportunities may be poor, growth rates might be expected to be relatively slow. A study of captive hawksbill turtles (Witzell 1980) demonstrated that neonate hawksbill turtles can grow as fast as 17 cm year^−1^, but no other studies exist to inform on growth rates for this life stage. Satellite-tracking studies have shown that turtles larger than 60 cm CCL may forage at depth in neritic waters, perhaps on large barrel sponges found in the ‘sponge belt’ at 80–120 m (Ghiold et al. 1994; Blumenthal et al. 2009b; Hawkes et al. 2012). This would largely preclude their capture using standard snorkel transect, free-diving hand capture, or SCUBA capture techniques to estimate growth rates. Carapace measurements recorded for successive nesting by females, however, may provide some insight into adult growth rates (e.g., 0.4 cm year^−1^ reported in (Beggs et al. 2007)), but these do not inform on adult males or large juveniles channeling resources only to somatic growth. Data are thus insufficient to derive a robust estimate of age to maturity, but a coarse minimum estimate could be generated from our model. Using the model data from the present study, a hatchling hawksbill turtle in the British Virgin Islands could reach sexual maturity at 67 cm CCL in <10 years (7 years and 8 months). This finding, however, should be cautiously interpreted. Growth rates in the present study have been extrapolated from recapture intervals as short as 63 days, and the present study lacks the resolution to assess whether there were seasonal variations in growth rate (which might be expected as some reptile species grow faster in warmer months (Pérez and Escobedo-Galván 2009; Arendt 2011; Ligon et al. 2012)). Finally, it is likely that there is considerable variation in individual growth rates due to stochastic environmental and biotic conditions. Bjorndal et al. (2013) showed that even for captive green turtles raised under similar, controlled conditions, there were considerable differences in the age (8–12 years) and size (88–119 cm CCL) of individuals at sexual maturity. We observed marked variation in growth rates in the present study, and such a difference might be expected to be more marked for wild populations, which probably experience greater environmental variability.

Estimates of age to maturity vary for cheloniid marine turtle species, with ridley turtles (*Lepidochelys olivacea* and *L. kempii*) thought to mature most quickly, in just 10 years (Zug et al. 2006; Snover et al. 2007; Caillouet et al. 2011; Avens and Snover 2013). Snover et al. (2013) showed that Hawaiian hawksbill turtles mature in approximately 17 years, and Caribbean hawksbill turtle studies suggest that sexual maturity is reached at 20 years or more (Boulon 1994; Crouse 1999; Meylan and Donnelly 1999). Larger loggerhead (*Caretta caretta*) and green (*Chelonia mydas*) turtles may take 25 years to reach sexual maturity (Casale et al. 2009; Goshe et al. 2010; Piovano et al. 2011), and leatherback turtles (*Dermochelys coriacea*) are thought to mature in as little as 12 years (Heppell et al. 2003; Dutton et al. 2005; Avens and Snover 2013). Growth rates in the present study, however, suggest that hawksbill turtles in the British Virgin Islands may mature more quickly than many other marine turtle populations and warrants further investigation.

If Caribbean hawksbill turtles do indeed reach sexual maturity in less time than previously thought, this could have significant implications for population demography, suggesting that population recovery after exploitation could take place much faster than has been previously considered. This also has important ramifications for the historic debate surrounding the harvest and trade of hawksbill turtle products from the Caribbean (e.g., ‘tortoiseshell’ from carapace keratin (Carillo et al. 1999; Mrosovsky 2000; Campbell 2002; Mrosovsky 2003; Godfrey et al. 2007; Mortimer et al. 2007; Webb 2008; Moncada et al. 2012)), suggesting that fundamental parameters used in assessing the effects of harvest might have been unrealistic (Crouse 1999; Meylan and Donnelly 1999; Mills 2013b). Indeed, if hawksbill turtles do mature more quickly than previously thought, the replenishment of the Caribbean populations by recruiting individuals would take place more quickly and the effect of the harvest might be less marked. However, the present study does not provide sufficient information to reconstruct the demographic model for Caribbean hawksbills, and more research is required to assess if our results are replicated elsewhere in the Caribbean.

It is interesting to speculate on why growth rates in the present study may be particularly fast, understanding that this may also be the case elsewhere in the Caribbean. Firstly, the waters of Anegada, like much of the Caribbean, are warm and thermally stable (mean 27.6°C ± 1.3 SD, see also Chollett et al. (2012), although they are not the warmest or least variable in the Caribbean). Because marine turtles are ectothermic, metabolic rate, and thus growth rate, is determined by the environment and warmer temperatures thus foster faster rates of growth (Gillooly et al. 2001; Zuo et al. 2012). Secondly, Anegada is surrounded by an extensive and particularly shallow coastal shelf (790 km^2^ surrounding Anegada is <20 m deep; (Witt et al. 2010)), much of which also hosts coral reef and associated habitats which provides both shelter and foraging opportunities for turtles. Clearly, there are extensive shallow seas and reefs surrounding many other Caribbean island nations (e.g., 14 of the 36 Caribbean nations reported in Burke and Maidens (2004) have greater reef area than do the British Virgin islands), which are also likely very warm and thermally stable. It therefore seems very possible that rapid rates of growth may also be realized elsewhere (Fig. 1). Finally, the Caribbean population of hawksbill turtles has undergone a massive reduction since historic times (by more than two orders of magnitude (McClenachan et al. 2006)), while the total abundance of some Caribbean reef sponges has probably increased (McMurray et al. 2010; Pawlik 2011) and may increase still further with future climate change (Bell et al. 2013). It has not been shown, however, whether sponge species that are known to be consumed by hawksbill turtles have increased (e.g., *Spirastrella coccinea*,*Ricordea florida*,*Chondrilla caribensis*,*Myriastra kalitetilla*,*Geodia neptuni*; (Meylan 1988; Leon and Bjorndal 2002; Rincon-Diaz et al. 2011)). While Caribbean hawksbill turtles in the past (prior to 1900) are estimated to have consumed up to 83% dry mass of the total sponge biomass and annual growth in the Caribbean ref, today, they probably consume <0.1% meaning that a much greater abundance of prey may be available for today's hawksbill turtles (McClenachan et al. 2006). Thus, it seems reasonable to hypothesize that fast growth rates on the Anegada shelf are fostered by extensive, sheltered, suitable habitat at warm temperatures with abundant and diverse forage food and suggests that rapid rates of growth should have been found elsewhere in the Caribbean too.

Future studies in the Caribbean should seek to address whether these rapid rates of growth are reflected in hawksbill turtle populations elsewhere, for example, in the huge reef system off the southern coast of Cuba (Burke and Maidens 2004), where a major foraging population of hawksbill turtles is found (Moncada et al. 2012) and where the oceanographic environment and prey availability are likely similar. Such future work would ideally incorporate skeletochronological work into age estimation of Caribbean hawksbill turtles, which does not appear to have been carried out to date and which would help provide robust estimates of age at sexual maturity. It is also essential that growth rates for hatchling, prerecruitment juvenile, and “subadult” juvenile turtles are collected. Should the growth rates presented in the present study be realistic, our current understanding of the demography of the Caribbean hawksbill turtle (Crouse 1999) will require revision.
